# Hindcasting injection-induced aseismic slip and microseismicity at the Cooper Basin Enhanced Geothermal Systems Project

**DOI:** 10.1038/s41598-022-23812-7

**Published:** 2022-11-14

**Authors:** Taiyi A. Wang, Eric M. Dunham

**Affiliations:** 1 Department of Geophysics, Stanford University, Stanford, USA; 2 Institute for Computational and Mathematical Engineering, Stanford University, Stanford, USA

**Keywords:** Geophysics, Seismology

## Abstract

There is a growing recognition that subsurface fluid injection can produce not only earthquakes, but also aseismic slip on faults. A major challenge in understanding interactions between injection-related aseismic and seismic slip on faults is identifying aseismic slip on the field scale, given that most monitored fields are only equipped with seismic arrays. We present a modeling workflow for evaluating the possibility of aseismic slip, given observational constraints on the spatial-temporal distribution of microseismicity, injection rate, and wellhead pressure. Our numerical model simultaneously simulates discrete off-fault microseismic events and aseismic slip on a main fault during fluid injection. We apply the workflow to the 2012 Enhanced Geothermal System injection episode at Cooper Basin, Australia, which aimed to stimulate a water-saturated granitic reservoir containing a highly permeable ($k = 10^{-13} - 10^{-12}$ $\hbox {m}{^2}$) fault zone. We find that aseismic slip likely contributed to half of the total moment release. In addition, fault weakening from pore pressure changes, not elastic stress transfer from aseismic slip, induces the majority of observed microseismic events, given the inferred stress state. We derive a theoretical model to better estimate the time-dependent spatial extent of seismicity triggered by increases in pore pressure. To our knowledge, this is the first time injection-induced aseismic slip in a granitic reservoir has been inferred, suggesting that aseismic slip could be widespread across a range of lithologies.

## Introduction

It is well known that fluid injection into fault zones can produce microseismicity and even damaging earthquakes^1–3^. Only in the past decade, however, has it been recognized that injection can also induce aseismic slip^4–6^. While shaking from injection-related earthquakes can damage well field equipment and buildings, aseismic slip can also have serious impact by shearing and damaging well casing^7^, an undesirable outcome for wastewater disposal and reservoir stimulation operations^8^. Even as theories of injection-induced seismic/aseismic slip emerge from numerical and laboratory studies^9–11^, validation of theories on the field scale is hindered by the dearth of aseismic slip observations.

Nonetheless, there is mounting evidence for aseismic slip during injection operations. In the southern Delaware Basin, Texas, for example, InSAR-derived surface deformation is well matched by $\sim$20 cm of aseismic slip on conjugate normal faults^6^ in a $\sim$1-km-thick unit comprised of sub-arkosic sandstones and siltstones. Modeling of injection-induced pressure diffusion, confined to a permeable fault damage zone, and slip on a fault with velocity-strengthening rate-and-state friction, helps constrain the fault zone fluid transport properties and pressure rise required to reproduce the observed aseismic slip^12^. In-situ injection experiments in carbonates have also produced aseismic slip on faults, with laboratory experiments and modeling used to constrain frictional and fluid transport properties^9,13,14^. In the Sichuan Basin, China, prevalent well casing deformation is attributed to aseismic fault slip associated with hydraulic fracturing treatments, which has significantly hindered shale gas production^8^. Hydraulic fracturing in the Montney Formation, British Columbia, Canada, has been linked to two large aseismic slip events ($M_w$ 5.0 and 4.2) on shallowly dipping thrust faults, based on InSAR-measured surface deformation^15^. Geothermal power extraction at the Brawley Geothermal Field, California, has been linked with earthquake swarms preceded by aseismic slip in sedimentary rocks^5,16,17^. These observations indicate that injection-induced aseismic fault slip may be a prevalent, yet potentially underdetected phenomenon.

**Figure 1:**
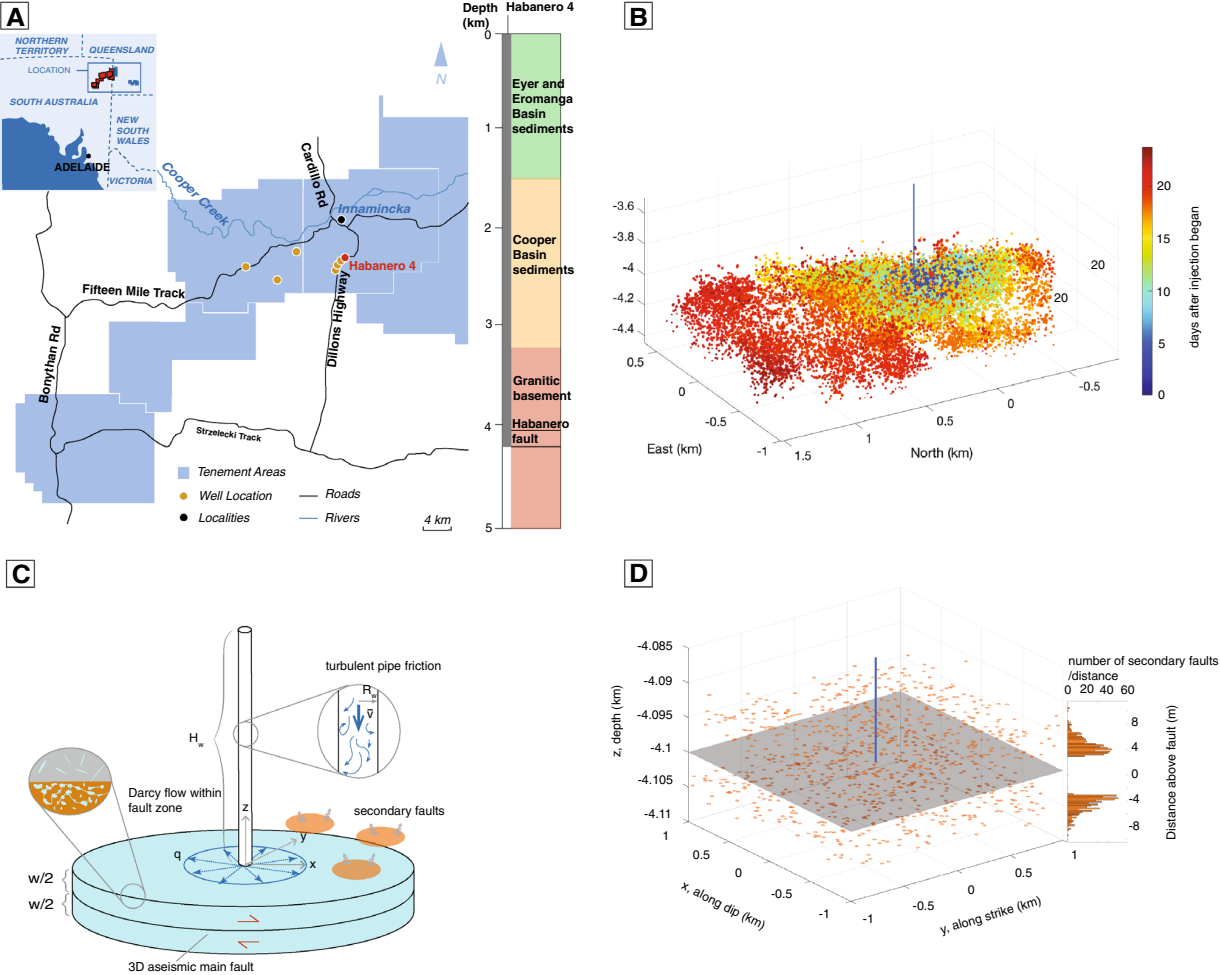
(**A**) Location of the Habanero Enhanced Geothermal System (EGS) field, South Australia (modified from a previous study^22^), and rock units along the depth of well Habanero 4 (adapted from a 2D resistivity model^24^). (**B**) Location of microseismicity from November 2012 injection episode at well Habanero 4. Colors indicate days after injection began. (**C**) Schematic of the model, which includes a 3D rate-and-state velocity-strengthening main fault (slip in *x* direction), discrete, velocity-weakening, secondary faults, and an injection well. The fault zone is of width *w*, within which fluid flow is described by radial Darcy flow. Pressure loss from turbulent pipe friction is accounted for when computing wellhead pressure from well bottom pressure. (**D**) Spatial distribution of rectangular secondary faults. The vertical locations of secondary faults follow a Gaussian distribution from the boundaries of the fault zone. Secondary faults are randomly distributed in the fault-parallel directions

To comprehensively assess the prevalence of aseismic slip, it is important to retrospectively identify and estimate aseismic slip during past fluid injections involving fault zones. This is particularly challenging when geodetic or borehole measurements are unavailable. The Enhanced Geothermal System (EGS) experiments in Cooper Basin, Australia, represent such challenges. EGS-related hydraulic stimulation experiments were performed in Cooper Basin from 2002 to 2013 (Fig. 1A)^18–21^. A total of 6 wells were drilled into water-saturated granites at depths of 3629 to 4852 m. Between 4077 and 4263 m below the surface, four injection wells penetrated a pre-existing thrust fault, later named the Habanero fault, which is outlined by injection-induced microseismicity (Fig. 1B) and dips slightly towards the west (strike $204^{\circ }$, dip $\sim$10$^{\circ }$). The sub-horizontal fault forms a brine reservoir with a pore pressure of $\sim 73$ MPa (significantly above the hydrostatic pressure, which is $\sim$40 MPa at the fault depth). Acoustic image logs show a $\sim$6 m thick pervasively fractured damage zone (hereafter referred to as the “fault zone”) surrounded by relatively impermeable granite. The presence of a highly permeable fault zone resulted in a series of failed attempts at producing the distributed permeability enhancement through fracturing and shearing that is expected in reservoir stimulation operations^22^.

From 2002 to 2013, injection experiments induced $\sim$75,000 earthquakes between $M_L$ 2.3 and 3.7, many of which concentrate around the Habanero fault. However, the possibility of aseismic slip, in light of its recognition in shale, carbonate, and sandstone reservoirs, has not been evaluated. In this study, we investigate the possibility that aseismic slip, in addition to seismic slip, occurred during the Nov. 2012 injection episode at the Habanero 4 well (17 days in duration, Fig. 1A). Aseismic slip monitoring was not present at the time, and the section of well intersecting the Habanero fault is uncased^23^, preventing direct indications of aseismic slip. The alternative possibility is that all slip was seismic and occurred on the Habanero fault in a swarm-like manner that tracked the advancing pressure diffusion front. This hypothesis was investigated in prior work^21^. We approach the problem with a workflow combining parameter calibration with conventional monitoring data and numerical modeling. The model accounts for fluid transport and pressure diffusion confined to a high permeability fault zone, aseismic slip on a main fault, and discrete microseismic events outside the fault zone (Fig. 1C,D). By matching the observed wellhead pressure and spatial-temporal distribution of microseismic events, we find that, subject to reasonable assumptions about the initial stresses and friction, aseismic slip likely contributed to half of the total moment release. Additionally, we find that direct fault weakening due to pore pressure increase, instead of elastic stress transfer (Coulomb stress changes) from aseismic slip, caused the majority of the observed microseismicity, although aseismic slip becomes the dominant triggering mechanism towards the end of the injection period. Finally, we introduce a simple theoretical model for predicting the space-time evolution of microseismicity in response to pore pressure diffusion that accounts for the initial stress and strength of the secondary faults.

## Workflow to hindcast injection-induced aseismic and seismic slip

Our numerical modeling workflow consists of four components: the well, fluid flow and pressure diffusion in the fault zone, aseismic slip on the main fault at the center of the fault zone, and microseismicity on secondary faults outside of the fault zone (see Methods). Wellhead pressure is computed from bottom hole pressure by correcting for the hydrostatic pressure difference and pressure loss from turbulent pipe friction. Fluid flow in the fault zone is represented by radial Darcy flow outward from the well. We model the Habanero fault (which we also refer to as the “main fault”) with velocity-strengthening (VS) rate-and-state friction. Secondary faults (which slip in microseismic events) are represented with the spring-slider model with velocity-weakening (VW) rate-and-state friction, and are randomly distributed within a 1 km $\times$ 1 km square box in the fault-parallel directions centered on the well. The fault-normal locations of the secondary faults relative to the main fault are sampled from a Gaussian distribution centered on the boundaries of the fault zone, with a standard deviation of 2 m. Secondary faults are oriented parallel to the main fault, which is informed by the predominance of low-angle thrust fault mechanisms of seismicity induced during the 2012 episode^25^.

The model is based on various assumptions regarding the nature of the fault system. We assume a velocity-strengthening main fault and velocity-weakening secondary faults. Prevalent microseismicity and the absence of a large earthquake on the main fault, given up to 15 MPa of pore pressure increase, as well as initial shear/normal stresses, suggest unconditionally stable sliding of the main fault (additional lines of reasoning in Discussion). Based on observed seismic event magnitudes (median $M_w = 0.6$; see Results for estimated fault dimension), we assume that seismogenic secondary faults are much larger than the imaged micro-fractures populating the fault zone around the main fault^22^. Therefore, all secondary faults are placed outside of the 6 m wide fault zone. We assume negligible stress interactions between secondary faults, which is justified when fault dimensions are small compared to distances between faults^26^. We also neglect stress perturbation feedback onto the main fault from the off-fault seismicity, which is supported by numerical experiments showing negligible elastic stress transfer from off-fault aftershocks to the main fault in a similar modeling study^27^. The last two assumptions allow for the efficient simulation of hundreds of microseismic events (Methods).

**Figure 2:**
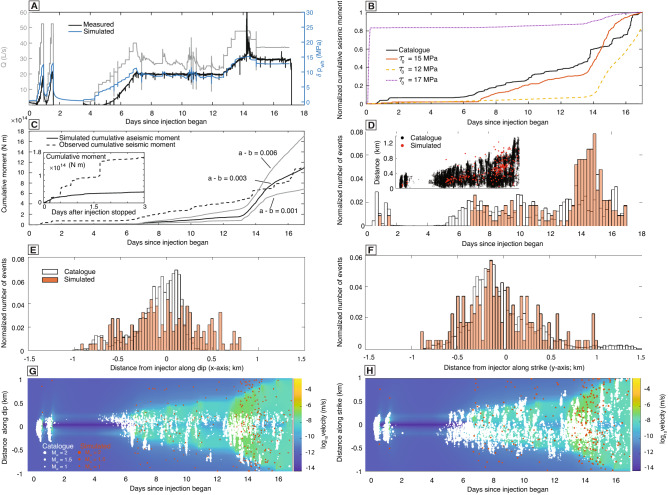
(**A**) Injection rate and wellhead pressure at Habanero 4 from Nov. to Dec. 2012. (**B**) Simulated cumulative moment due to seismic slip for initial shear stress of 12, 15, and 17 MPa, as well as cumulative moment derived from seismic catalog. Both are normalized by total moment. (**C**) Cumulative seismic moment derived from microseismicity catalog and cumulative aseismic moment from simulation. Simulated cumulative aseismic moment is shown for different $a-b$. Inset shows the cumulative seismic/aseismic moment after injection stopped (note that cumulative moment axis is a factor of 10 smaller). (**D**) Number of seismic events per time interval normalized by total number of events. Inset shows the migration of seismicity away from the well. (**E**), (**F**) Number of seismic events per distance interval along dip and strike, respectively, normalized by the total number of events. The events along each axis are restricted to within 100 meters from the well along the other axis. (**G**), (**H**) Time evolution of seismicity (dots) and main fault slip velocity (color), along dip and strike, respectively. All results are for an initial shear stress of 15 MPa and main fault $a - b = 0.003$, unless otherwise noted

When calculating pressure change within the fault zone, we assume radial Darcy flow and linear pore pressure diffusion, neglecting leak-off into the relatively unfractured granite outside the fault zone. Although it was suggested^22^ that injection-induced shear slip on the Habanero fault enhanced permeability, *k*, and porosity, $\phi$, in a later stimulation period (Oct. to Dec. 2013), our modeling suggests that slip-induced permeability and porosity enhancement are not required to explain wellhead pressure of the 2012 injection period (Fig. 2A). Therefore, we neglect porosity and permeability enhancement. We assume uniform pore pressure across the width of the fault zone, which is justified because the injection time scales of interest (hours to days) are much longer than the diffusion time across the fault zone width ($t_D = w^2 /\alpha < 10$ s; *w*: fault zone width; $\alpha = k/(\phi \eta \beta )$, diffusivity; $\eta$: fluid viscosity; $\beta$: sum of fluid and pore compressibility; values are in Table 1). Leak-off outside the fault zone is neglected when calculating fault zone pressure changes following the arguments in Appendix B of reference^11^. However, we do account for pressure changes on secondary faults surrounding the fault zone, which we later argue is required to induce most of the microseismic events. As a proxy for leak-off driven pressure changes, we extend the fault zone pressure changes outward with a decaying Gaussian function having 2 m standard deviation, the value chosen as an estimate of the diffusion length over a duration of 1 hour using permeability $k = 10^{-16}$ m$^2$ for jointed granite^28^, the other parameters being the same as in the previous scaling estimate.

Our workflow takes injection volume rate as input, and produces as output the fault zone pressure, wellhead pressure, aseismic slip and stress evolution on the main fault, and slip and stress evolution on the secondary faults. First, the fault zone pressure diffusion equation is solved using the known injection rate as the input source. The fault zone permeability *k* is treated as a calibration variable. Second, the fault zone pressure history is used as input to solve for aseismic slip on the main fault, given fixed frictional parameters and calibrated initial shear stress (with the calibration performed in the subsequent step by matching microseismicity). Third, the stress perturbations from aseismic slip and fault zone pore pressure are used to drive seismic events off the fault, given an assumed fault patch stiffness and event locations. Fixed and calibrated model inputs are listed in Table 1.

We first calibrate the permeability *k* of the fault zone by tuning it to match the simulated and observed wellhead pressure history, using the injection rate as input and assuming a nominal porosity of $\phi = 1 \%$ (similar to the $1.4 \%$ porosity derived from tracer tests^29^). We then calibrate the initial shear stress (which is the same on the main fault and the secondary faults, given their proximity and identical orientation) by matching the simulated and observed normalized cumulative seismic moment. This calibration is required due to uncertainties in local stress conditions. Constraints on reservoir stress are provided through a combination of geomechanical modeling and logging/drilling data analysis. Integration of density log data as a function of depth yields an overburden stress, $S_v$, of 100 MPa at $\approx 4100$ m, where Habanero 4 intersects the fault^22^. Modeling of borehole breakout and tensile fracture data indicates a maximum horizontal stress, $S_{Hmax}$, of 150 MPa and a minimum horizontal stress, $S_{hmin}$, of 120 MPa^22,30,31^. Estimates of horizontal stress are contingent on assumptions of rock strength and therefore have significant uncertainties. Resolving $S_v$ and $S_{Hmax}$ onto a 10$^{\circ }$ dipping fault results in a normal stress, $\sigma _0$, of 101.8 MPa, and a shear stress, $\tau _0$, of 10.3 MPa. Together with an estimated initial pore pressure, $p_0$, of 73 MPa^22^, this gives $\tau _0/(\sigma _0-p_0) = 0.36$, much less than the typical granite friction coefficient of 0.6. Our modeling shows that $\tau _0 = 10.3$ MPa is too small to produce slip on the secondary faults and is therefore deemed incompatible with the observed seismicity. Therefore, we fix $\sigma _0$ to 101.8 MPa and calibrate $\tau _0$ following an iterative procedure. First, an initial shear stress is chosen for both the main fault and secondary faults. The fault zone pressure derived with the calibrated permeability is used to drive aseismic slip on the main fault, without coupling to secondary faults. The fault zone pressure history and the elastic stress changes from aseismic slip are then used to drive off-fault microseismicity. Following this procedure, we adjust the initial shear stress to match the simulated and observed cumulative seismic moment (normalized by maximum cumulative seismic moment to account for the smaller number of secondary faults used in our simulation as compared to reality). Our hindcast for aseismic and seismic slip is done using the calibrated hydraulic permeability and initial shear stress.

## Results

### Calibrated fault zone permeability and initial shear stress

**Figure 3:**
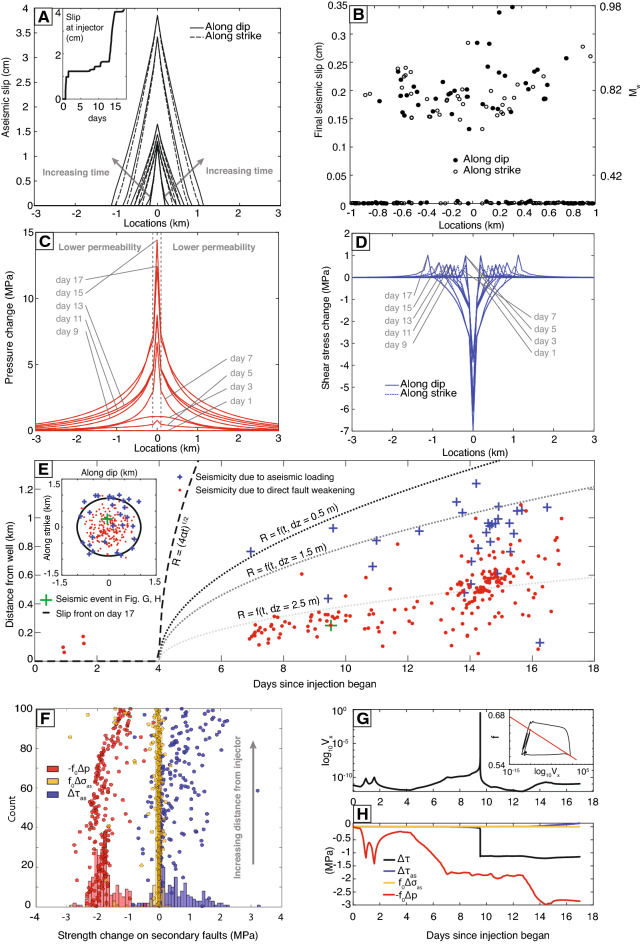
Time evolution of (**A**) aseismic slip on the main fault along dip and strike directions. Inset shows cumulative slip at the well. (**B**) Total seismic slip at the end of injection. (**C**) Pore pressure change in the fault zone. Vertical dashed gray lines mark the transition from the higher permeability near-well region to the lower permeability region. (**D**) Shear stress change on the main fault. Aseismic slip, pressure, and shear stress change are plotted every 2 days. (**E**) Space-time plot of the simulated seismicity and mechanisms that induced them. Inset shows map view of seismicity. Dashed line shows the diffusion length scale over time; dotted lines show the theoretical prediction of seismicity front triggered by pore pressure change (Methods). *t*:  time, *dz*:  vertical distance of secondary faults from the main fault. (**F**) Histogram showing contributions to fault strength change on secondary faults at the time of a seismic event (slip velocity > 0.1 m/s): direct weakening from pressure increase, $-f_0 \Delta p$; shear stress loading from aseismic slip, $\Delta \tau _{as}$; normal stress loading from aseismic slip, $f_0 \Delta \sigma _{as}$. Dots show contributions of each mechanism as a function of cumulative counts of seismicity. (**G**) Secondary fault slip velocity evolution (at location marked in (**E**)). Inset shows friction coefficient versus slip velocity. (**H**) Contribution of main fault aseismic slip and pore pressure diffusion to inducing seismic slip at the secondary fault location marked in (**E**). $\Delta \tau$: change in total shear stress.

Calibration of fault zone permeability and initial shear stress improves the accuracy of the aseismic/seismic slip hindcast. We first calibrate the permeability, *k*, of the fault zone. We find higher *k* near the well is required to fit the observed wellhead pressure (Fig. 2A), which may reflect permeability enhancement due to repeated prior stimulations. The best-fit *k* for the Habanero Fault in Nov. 2012 is $1.1 \times 10^{-12}$ $\hbox {m}{^2}$ within 150 m of the well and $4 \times 10^{-13}$ $\hbox {m}{^2}$ beyond 150 m from the well. Our values of permeability are similar to in-situ closed loop injection calibrated permeability of $7.9\times 10^{-13}$ to $3.9\times 10^{-12}$ $\hbox {m}{^2}$^29^. A higher permeability near the well is required to match wellhead pressure data within the first two days of injection, when the pressure perturbation is limited to a few hundred meters from the well (Fig. 3C). If the near-well permeability is the same as the lower permeability away from the well, the model overpredicts the amplitude of the pressure peaks in days 1 and 2 (Fig. 2A). A lower permeability away from the well is simultaneously required. If the permeability away from the well is the same as the higher permeability near the well, the model underpredicts the amplitude of the pressure plateaus during days 7 to 17. We note that a similarly sized region of enhanced permeability was found for the 2003 stimulation of the nearby Habanero 1 well^32^.

**Table 1:** Parameters used for hindcasting injection induced seismic and aseismic slip

Parameter	Symbol	value
**Fault model**		
Main fault dimensions		$20 \times 20$ km
Secondary faults dimensions		$20 \times 20$ m
Fault width	*w*	6 m
Shear modulus$^{1}$	$\mu$	24 GPa
Poisson’s ratio	$\nu$	0.25
Main fault direct and state evolution effect parameters	$a_{as}$, $b_{as}$	0.015, 0.012
Secondary fault direct and state evolution effect parameters	$a_{ss}$, $b_{ss}$	0.015, 0.018
Reference velocity	$V^*$	1 $\times$ $10^{-6}$ m s$^{-1}$
Reference friction coefficient	$f^*$	0.6
State evolution distance	$d_c$	0.0153 mm
Radiation damping coefficient	$\eta _{rad}$	4.55 MPa s m$^{-1}$
Main fault normal stress$^{2}$	$\sigma$	101.8 MPa
Initial pore pressure$^{3}$	$p_{0}$	73.8 MPa
Initial shear stress	$\tau _0$	15 MPa
Initial dimensionless state variable	$\Psi _{0}$	0.75
Standard deviation for secondary faults density (in vertical direction)		2 m
Standard deviation for interpolating pressure to secondary faults (in vertical direction)		2 m
**Fluid model**		
Fluid density	$\rho$	1000 kg m$^{-3}$
Permeability within 150 m from well	$k_{near}$	$1.1 \times 10^{-12}$ $\hbox {m}{^2}$
Permeability beyond 150 m from well	$k_{far}$	$4 \times 10^{-13}$ $\hbox {m}{^2}$
Nominal fault rock porosity	$\phi$	0.01
Fluid viscosity	$\eta$	$8 \times 10^{-4}$ Pa s
Sum of pore^33^ and fluid compressibility^34^	$\beta$	$1 \times 10^{-8}$ $\hbox {Pa}{^{-1}}$
**Habanero 4 well**		
Depth to fault$^{4}$	$H_{w}$	4077 m
Tubing inner diameter $^{4}$	$D_{w}$	17.8 $\hbox {cm}$
Darcy-Weisbach pipe friction factor$^{5}$	$f_{D}$	0.015

[1] Shear modulus of granite^35^ [2] Derived from $S_{Hmax}$ and $S_v$, which are obtained from geomechanical modeling^22^ [3] From pressure build up tests^22^ [4] Well completion report^23^ [5] Moody diagram^36^.

We then calibrate the initial shear stress, $\tau _0$, on the main fault and secondary faults. The closer secondary faults are to being critically stressed, the more seismicity occurs at the beginning of injection period. A lower initial shear stress results in more seismicity occurring at the end of injection period. We find that $\tau _0 = 15$ MPa, higher than $\tau _0 = 10.3$ MPa implied by the estimated $S_{Hmax}$ and $S_v$, is required to explain the observed seismicity. The choice of $\tau _0$ on the secondary faults strongly influences the temporal distribution of seismic events, as well as the temporal trend of total seismic moment release (Fig. 2B). Because the simulated seismicity rate depends on the number of secondary faults, which for computational reasons is much fewer than the observed number of events, we focus our comparison on the temporal trend of cumulative seismic moment, normalized by the cumulative seismic moment at the end of injection. We use $\tau _0/f^{*} \sigma _0^{'}$ as the proxy for closeness-to-failure, where $f^* = 0.6$ is the reference friction coefficient at reference velocity $V^* = 1 \times 10^{-6}$ $\hbox {m s}{^{-1}}$, representative of aseismic slip velocities, and $\sigma _0^{'}=\sigma -p_0$ is the initial effective normal stress. At $\tau _0 = 10$ MPa ($\tau _0 / f^{*}\sigma _0^{'} \approx 0.6$), no seismicity (slip velocity > 0.1 m/s) is induced. At $\tau _0 = 12$ MPa ($\tau _0 / f^{*}\sigma _0^{'} \approx 0.7$), most events occur after day 14, inconsistent with the observations (Fig. 2B). At $\tau _0 = 15$ MPa ($\tau _0 / f^{*}\sigma _0^{'} \approx 0.9$), the simulated cumulative seismic moment release best fits the observations. At $\tau _0 = 17$ MPa ($\tau _0 / f^{*}\sigma _0^{'} \approx 1$, the critically stressed fault condition), seismic events occur immediately after injection starts, also inconsistent with the observations.

The temporal pattern of seismicity depends on both $\tau _0$ and the stability of secondary faults, but the uncertainty in the latter does not significantly impact the calibration of $\tau _0$. Fault stability in the spring-slider model is controlled by the non-dimensional ratio $\kappa /\kappa _{critical}$. The fault stiffness (shear stress change per unit slip) $\kappa$ scales with $\mu /H$, where $\mu$ is the host rock shear modulus and *H* the fault dimension^37^. The fault dimension *H* is chosen to approximately match the predicted moment magnitudes of seismicity with those of the observations ($M_0 \sim H^3 \Delta \tau$; for co-seismic stress drop $\Delta \tau = 1$ MPa, median seismic moment $M_0 = 1 \times 10^{10}$ $\hbox {N.m}$, $H \sim 20$ m) and $\mu$ is chosen as a typical laboratory-measured granite shear modulus^35^. There is more uncertainty in the critical stiffness, $\kappa _{critical} = \sigma ^{'}(b - a)/d_c$, where $d_c$ is the state evolution distance. The fault is unstable (seismogenic) when $\kappa /\kappa _{critical} \ll 1$. For simulated secondary faults, $\kappa \approx 1.2 \times 10^{9}$ $\hbox {Pa.m}{^{-1}}$ ($H = 20$ m, $\mu = 24$ GPa), and $\kappa _{critical} \approx 5 \times 10^9$ $\hbox {Pa.m}{^{-1}}$, yielding $\kappa /\kappa _{critical} = 0.24$. At Cooper Basin, the abundance of off-fault seismicity indicates $\kappa /\kappa _{critical} \ll 1$ for the secondary faults. Therefore, uncertainties in $d_c$ or $b-a$ will influence $\kappa _{critical}$ used for simulation, but, within a plausible range, will not qualitatively influence the simulated temporal pattern of seismicity.

### Injection-induced aseismic and seismic slip

We find that at the end of the injection period, a maximum (simulated) aseismic slip of 4 cm occurs where the fault intersects the well, with cumulative slip decreasing to zero approximately 1.2 km away from the well (Fig. 3A). The spatial extent of aseismic slip is close to axisymmetric but extends slightly further along the dip direction as expected theoretically^38^. During the initial two peaks (days $1 - 2$) in injection rate (Fig. 2A), about 1.5 cm of aseismic slip occurs near the well (Fig. 3A). During the pause in injection (days 2–4), no additional aseismic slip occurs. When injection again increases (days 5–7), slip remains very slow. During nearly constant rate injection at 25 $\hbox {L s}{^{-1}}$ (days 8–13), slip increases gradually. When injection rate increases again (days 13–14), slip accelerates (Fig. 2G,H). Finally, when injection rate plateaus at 40 $\hbox {L s}{^{-1}}$ (day $14 - 17$), slip continues increasing, but at a slower rate.

Maximum seismic slip is about 0.35 cm, with larger seismic slip closer to the injection well (Fig. 3B). The simulated seismicity matches the spatial-temporal distributions of observed seismicity remarkably well (Fig. 2D–F). In addition, the simulated seismicity migrates away from the well at the same rate ($\sim$50 $\hbox {m day}{^{-1}}$) as the observed seismicity (Fig. 2D inset). Similar to previous injection modeling in 2D^39^, we find that there is a clear correlation between the rate of pressure increase (indicated by wellhead pressure in Fig. 2A) and seismicity rate (Fig. 2D).

The estimated minimum value for cumulative aseismic slip at the well is 2 $\hbox {cm}$, accounting for uncertainties in frictional parameters (Fig. 2C). On the main fault, as long as the state evolution distance, $d_c$, is much smaller than the total slip, most of the slip occurs when *f* is evolving along the steady state $f_{ss} = f^* + (a-b) \log (V/V^*)$^11^. This suggests that, $a-b$ influences the magnitude of total slip, if $V/V^*$ is not close to unity. Because we chose $V^* = 10^{-6}$ $\hbox {m s}{^{-1}}$ to be representative of the modeled aseismic slip velocity, $a-b$ has relatively small influence on total slip. Holding all input parameters the same, $a - b$ of 0.001 and 0.006 (with *a* fixed to 0.015) results in 2 and 5 $\hbox {cm}$ of slip at the well, corresponding to cumulative aseismic moment of $7 \times 10^{14}$ $\hbox {N m}$ and $17 \times 10^{14}$ $\hbox {N m}$, respectively (compared to $11 \times 10^{14}$ $\hbox {N m}$ for the preferred model, Fig. 2C). Because the slip velocity inside the crack is slightly lower than $V^*$, steady state friction is lower for higher $a-b$; higher $a-b$ produces larger slip due to increase in stress drop along steady state^11^.

How robust is the minimum value for cumulative aseismic slip at the well with respect to choice of initial state? In general, moderate variations in initial state should not result in large variations in aseismic slip magnitude^12^. Increasing the dimensionless initial state $\Psi _0 = f^{*} + b \log (\theta V^{*}/d_c)$ ($\theta$ being the usual state variable having units of time) delays the onset of significant slip and reduces the total amount of slip. $\Psi _0$ is approximately bounded by examining its corresponding peak or “static” friction coefficient, $f_s$, defined as the maximum friction coefficient value prior to the onset of state evolution and drop in friction. For our choice of $\Psi _0 = 0.75$, $f_s$ reaches 0.72, which is comparable to typical static friction values for granite. Further increasing the initial state will only make the static friction higher. Therefore, we argue that, any reasonable choice of initial state would result in cumulative slip equal to or higher than the current values, holding all other parameters constant.

## Discussion

### Aseismic moment release accounts for half of total moment release

Given the inferred fault zone permeability and initial shear stress, our model quantifies the amount of aseismic and seismic slip, which we discuss here in terms of moment release (area $\times$ average slip $\times$ shear modulus). We find that aseismic moment likely contributed to half of the total moment by comparing the simulated cumulative aseismic moment with the observed cumulative seismic moment (Fig. 2C). The simulated aseismic moment is compared to the observed seismic moment, rather than to the simulated seismic moment, because the simulated seismic moment depends on the assumed number of secondary faults (Methods). Our finding of aseismic moment accounting for half of the total moment has practical implications for injection operations. While aseismic slip could in theory enhance fault zone permeability, thereby benefiting stimulation operations^4,40^, it might also shear and deform cased wells. This can jeopardize well stability and hinder fluid flow and the deployment and retrieval of wellbore tools^8^, reducing the economic viability of the operations.

In contrast to our finding, previous work assumed that all slip on the Habanero fault was seismic^21^, and inferred up to 25 cm of slip at the well from the total seismic moment release (assuming an average stress drop of 0.1 MPa and a shear modulus of 12 GPa). Independent validation of such predictions is difficult, because the section of Habanero 4 intersecting the fault zone is uncased. Multiple lines of evidence, however, support the conceptual model of a velocity-strengthening main fault and velocity-weakening secondary faults. First, in hydrothermal environments, the preferential flow of hydrothermal fluid through the fault zone can form phyllosilicates, a mineral group known for promoting stable fault sliding^41,42^. Hydrothermal fluid can form phyllosilicates through either weathering of the granitic host rock or precipitation. Phyllosilicates are expected to be less common on lower-permeability secondary faults away from the permeable fault core. Second, a well-developed fault fabric within the fault core may also promote stable sliding^43^, thereby favoring aseismic slip. Secondary faults typically have less developed fabric due to their smaller cumulative slip. Third, in-situ and laboratory experiments indicate that pressurized, water-saturated fault gouge tends to be velocity-strengthening when slipping^9^. Seismic observations at tectonic faults^44^ and numerical experiments^27^ show an abundance of off-fault aftershocks, suggesting that secondary faults surrounding the main fault tend to be velocity-weakening.

Our model predicts that accelerations of aseismic slip are preceded by an increase in seismicity. This is in contrast to cases where seismicity is inferred to have been caused by aseismic slip, where aseismic fault slip precedes a large increase in the number of seismic events^13^. For Habanero 4, our model suggests that most of the aseismic moment release occurred during the last four days of the injection period (days 14 to 17) when injection was sustained at a high rate. Until day 13, aseismic moment is less than $20 \%$ of the seismic moment. The rapid increase in aseismic moment release after day 13 is due to the large magnitude of fault strength decrease from an increase in pore pressure (Fig. 3C). After injection stops, both aseismic and seismic moment release rate drop immediately, indicating a short lag time between injection and slip. This is similar to findings from previous numerical models of injection-induced aseismic slip in the Delaware Basin^12^. By the third day after injection stops, the aseismic and seismic moment stops increasing. Cumulative seismic moment increased four times as much as the aseismic moment during the post-injection period.

### Fault weakening due to pore pressure changes drives microseismicity

Is microseismicity induced by direct fault strength change from pore pressure increase ($-f^* \Delta p$) or by elastic stress transfer from aseismic fault slip (shear stress change: $\Delta \tau _{as}$, strength change from normal stress change: $f^* \Delta \sigma _{as}$)? We find that the dominant mechanism for triggering microseismicity is weakening of fault strength from pressure increase, except at sufficiently large distance from the well, where elastic stress transfer from aseismic slip becomes more important as the pressure increase becomes smaller.

On average, the simulated magnitude of $-f^* \Delta p$ at secondary faults exceeds $\Delta \tau _{as}$ and $f^* \Delta \sigma _{as}$ (histogram in Fig. 3F; example time series and corresponding velocity history in Fig. 3G,H). However, beyond $\sim$0.5 km from the well, shear stress increase from aseismic slip become larger than the reduction in strength from the pressure increase (Fig. 3D–F), resulting in aseismic loading being the dominant mechanism. This threshold distance at which aseismic loading becomes the dominant triggering mechanism is controlled by both the initial closeness to failure of the fault^45,46^ and the injection history. For constant rate injection, the degree by which the slip front outpaces the pressure front is dictated by the dimensionless aseismic slip amplification number, $\lambda$^10,38^. The dimensionless aseismic slip amplification number is related to the stress injection parameter, which is $(1 - \frac{\tau _0}{f \sigma _0^{'}}) \frac{\sigma _0^{'}}{\Delta p_*}$, where $\Delta p_* = \frac{{\bar{Q}} \eta }{4 \pi k w}$ (${\bar{Q}}$: constant volume injection rate) is the characteristic pressure and *f* a representative frictional coefficient. Between days 4 and 13, we can approximate the injection history as a step function. For this period, the stress injection parameter in our preferred model is $\sim$4.5 (${\bar{Q}} = 25$ $\hbox {L s}{^{-1}}$, $\eta = 8 \times 10^{-4}$ $\hbox {Pa s}$, $k \approx k_{far} = 4 \times 10^{-13}$ $\hbox {m}{^2}$, $f = 0.6$, $w = 6$ m, $\sigma _0^{'} = 28$ $\hbox {MPa}$, $\tau _0 = 15$ $\hbox {MPa}$), with corresponding $\lambda$ of 0.1, implying a slip front propagating slower than the pressure front. While the solution for $\lambda$ as a function of fault stress parameter does not account for the time dependence of the actual injection history, $\lambda$ scales with $\sqrt{\Delta p_{*}} \propto \sqrt{{\bar{Q}}}$, in the critically stressed asymptotic limit^38^. Therefore, the subsequent increase in injection rate on day 13 (Fig. 2A), and the corresponding increase in fault zone pressure (Fig. 3C), enables the slip front (and the accompanying seismicity induced by aseismic loading) to outpace the pressure front. This observation highlights the role of injection history in controlling the relative contribution of fault weakening and aseismic stress transfer to inducing seismicity.

The classic diffusion length scale $\sqrt{4 \alpha t}$ over-predicts the spatial extent of seismicity at any given time (Fig. 3E), because it does not account for the reduction in strength that is required to bring a fault that is not critically stressed to the failure condition. Furthermore, when the secondary faults are outside of the high permeability fault zone through which pressure diffusion is approximately confined, the pressure outside that fault zone on the secondary faults is reduced relative to its value in the fault zone. We introduce a simple theoretical model (specific to a step function injection rate history) that accounts for these and provides as an output the maximum radial distance from the injection well where conditions to trigger seismicity are met (Methods). In applying this model to Habanero 4, we again approximate the injection history between days 4 and 13 as a step function. The theoretical model for secondary faults at a vertical distance $dz = 2.5$ m from the main fault nicely delineates the spatial extent of simulated seismicity up until day 13 (Fig. 3E), when injection rate increases significantly. Given the same injection rate, the closer the secondary faults are to the main fault, *dz*, the farther the expected spatial extent of seismicity at any given time. In general, *dz* could be constrained through borehole imaging of fault plane distributions or relocated microseismicity. Therefore, the model provides a theoretical maximum distance at which seismicity can be induced by pore pressure change.

## Conclusion

We present a model relating injection rate history to wellhead pressure, main fault aseismic slip, and off-fault microseismicity. Using this model, we calibrate the permeability and stress state of the Habanero Fault. We infer that aseismic slip accounts for $\sim$40 to $60\%$ of the total moment release during the 2012 injection period, accounting for uncertainties in initial shear stress and fault frictional properties. We deduce that fault weakening due to pore pressure diffusion is the main driver of microseismicity up to 0.5 km from the Habanero 4 well, beyond which elastic stress transfer from aseismic slip on the main fault becomes the dominant mechanism. The dominance of fault weakening induced seismicity is due to both relatively low initial stress and the short duration of injection, which ended before elastic stress transfer becomes the dominant mechanism. The spatial extent of pore pressure change induced seismicity at a given time can be estimated using the proposed theoretical model. The estimated 4 cm of aseismic slip near the well, if occurring on a fault that crosses a cased well, could severely impact well stability. Our inference of significant aseismic slip in a region previously thought to only experience seismic slip suggests that aseismic slip could happen on faults hosted in granitic rocks. We acknowledge that there are no direct measurements that can be used to validate our model prediction of aseismic slip. It is therefore possible that the microseismicity data might instead be reflective of seismic slip on the main fault, which occurs in a swarm-like manner (concentrated near the advancing pressure diffusion front) because the fault only becomes critically stressed with sufficient pressure rise and stress transfer from prior seismic slip, as investigated previously^21^. Nonetheless, our modeling approach can be used to quantitatively investigate the possibility of aseismic slip at other reservoir stimulation locations where a seismic catalog and wellhead pressure records are the only observational constraints.

## Methods

### Fluid model

We first consider fluid transport and radial pore pressure diffusion integrated across the width of the fault zone. Accounting for both fluid and pore compressibility, but neglecting poroelastic effects from rocks outside of the fault zone^47^, we have the following diffusion equation for injection-induced pore pressure change, *p*(*r*, *t*):

1 $$\begin{aligned} 2 \pi r w \phi \beta \frac{\partial p}{\partial t} - \frac{\partial }{\partial r} \left( 2 \pi r w \frac{k}{\eta } \frac{\partial p }{\partial r} \right) = 0 \end{aligned}$$

where *r* is the radial coordinate, *w* is the fault zone width, $\phi$ is the nominal porosity, $\beta = \beta _f + \beta _{\phi }$ is the total (fluid + pore) compressibility, *k* is the fault zone permeability, and $\eta$ is the fluid viscosity. The time-dependent boundary condition at the well is

2 $$\begin{aligned} Q (R_w, t)= Q_{out} (t) = - 2 \pi r w \frac{k}{\eta } \frac{\partial p}{\partial r} \Big |_{r = R_w}, \end{aligned}$$

where $R_w$ is the well radius.

A 4th order SBP-SAT finite difference method^48^ is used for spatial discretization of the variable coefficient second derivative operator in Eqn. 1. Grid points are logarithmically distributed to ensure dense grid nodes near the well. The semi-discretized equation is then solved with MATLAB’s stiff ODE solver ode15s. We verify the accuracy of the diffusion solver by comparing the numerical solution with a known analytical solution^49^, derived for pressure perturbation due to constant rate injection into an infinitely long fault zone.

To relate the bottom hole pressure, $p_b (t) = p (r = R_w, t)$ to wellhead pressure, $p_h(t)$, we possibly need to consider the storativity of the well and pressure loss due to turbulent flow in the pipe. We neglect the effect of well storativity on pressure perturbations. This is justified by considering the following linearized form of the fluid mass in the well:

3a $$\begin{aligned} \frac{\partial p_w}{\partial t}&= \frac{Q_{net} }{\beta _w V_{w}} \end{aligned}$$

3b $$\begin{aligned} Q_{net}&= Q_{in} - Q_{out} \end{aligned}$$

where $p_w$ is the pressure perturbation in the well due to well storativity (which we assume is spatially uniform for this estimate), $\beta _w$ is the total well compressibility, and $V_w$ the well volume. The well compressibility $\beta _w$ is approximately equal to the fluid compressibility in a cased well, but requires a small correction involving the shear modulus of the formation for an uncased well^50^. The volumetric net flow, outflow, and injection rates are $Q_{net}$, $Q_{out}$, and $Q_{in}$, respectively. Because $p_w$ must match the bottom hole pressure perturbation on the fault, $p_b$, Eq. 3 can be re-arranged as $Q_{net} = \frac{\partial p_b}{\partial t}\beta _w V_w$. We run the fluid model neglecting well storativity, and use the resulting $p_b (t)$ to estimate $Q_{net}$. For the range of $p_b(t)$ in our simulation, $Q_{net}/Q_{in} \ll 1$. Therefore, $Q_{in} \approx Q_{out}$, and it is justified to neglect well storativity for Habanero 4.

We neglect pressure loss from near-well friction due to flow through geometrically complex fracture zone, but account for the pressure loss due to turbulent pipe flow^36,51–53^:

4 $$\begin{aligned} p_{pipe}(t) = f_D \frac{8 H_w \rho Q_{in}^2}{\pi ^2 D_w^5} \end{aligned}$$

where $f_D$ is the Darcy-Weisbach friction factor, $H_w$ is the well length, and $D_w$ is the well diameter. Therefore, the wellhead pressure, $p_h (t)$ can be related to well bottom pressure $p_b$ by correcting for pipe friction and hydrostatic pressure:

5 $$\begin{aligned} p_h(t) = p_b(t) + \rho g H_w + p_{pipe}(t). \end{aligned}$$

In contrast to well storativity, pressure losses from pipe friction cannot be neglected, especially during the high injection rate periods.

### Main fault model

We consider a 3D quasi-static problem with a planar fault embedded in a linear elastic full space (Fig. 1C). The full space assumption is justified, given that the aseismically slipping portion of the fault is of order 1 km, whereas fault depth $\sim 4$ km. The fault has uniform initial effective normal stress, $\sigma ^{'}_0 = \sigma - p_0$ (fault normal stress minus pore pressure), and shear stress, $\tau _0 = \sigma _{zx}$. We neglect rake rotation (i.e., changes in the slip direction, which is assumed to be exclusively in the *x* direction) and prohibit opening in *z* direction (coordinate system in Fig. 1C). The stress is related to the displacement field through Hooke’s law for an isotropic solid and the strain-displacement equations. Slip velocity is defined as

6 $$\begin{aligned} \quad V(x, y, t) = \frac{\partial \delta }{\partial t}, \end{aligned}$$

where $\delta$ is slip in the *x* direction. The physics of standard earthquake sequence modeling is completed with rate-and-state friction and an aging law for the state variable:

7a $$\begin{aligned} f(\theta , V)&= f^* + a \log \frac{V}{V^*} + b \log \frac{\theta V^*}{d_c}, \end{aligned}$$

7b $$\begin{aligned} {\dot{\theta }}&= 1 - \frac{\theta V^*}{d_c}, \end{aligned}$$

where *a*, *b* are the dimensionless direct effect and state evolution parameters; $V^*$ is a reference velocity; $f^*$ is a reference friction coefficient for steady sliding at $V^*$; and $d_c$ is the state evolution distance. Enforcing the friction law at the fault surface requires

8 $$\begin{aligned} \tau _0 + \Delta \tau - \eta _{rad} V = f(\theta , V) (\sigma _0^{'} - p), \end{aligned}$$

where $\Delta \tau$ is the shear stress perturbation due to aseismic slip and $\eta _{rad}$ is the radiation damping parameter. For a planar fault in a uniform full space, no normal stress change is induced by fault slip.

We solve the quasi-static 3D elasticity problem with a Fourier spectral method^54^. We verify the static elasticity solutions by using the slip due to uniform stress drop in a circular shear crack^55^ as an input to the spectral boundary element code, and comparing the shear stress drop within the crack to the known stress change. For the more general problem with rate-and-state friction, the slip $\delta$ and dimensionless state variable $\Psi = f^* + b \log \frac{\theta V^*}{d_c}$ are time stepped using an adaptive, explicit third order Runge-Kutta method with an embedded error estimate, with error control on $\delta$ and $\Psi$^56,57^.

### Secondary fault model

We use lumped parameter spring sliders to simulate microseismicity induced by fluid injection, neglecting interactions among spring sliders. The slip history of each spring slider is obtained by solving (using the same adaptive Runge-Kutta method) $d \delta / dt = V$, the state evolution equation, and

9 $$\begin{aligned} \tau _0 + \Delta \tau _{as} - K D(t)- \eta _{rad} V = f(V, \Psi ) (\sigma _0 + \Delta \sigma _{as} + \Delta \sigma _{s} - p(t)), \end{aligned}$$

where $\kappa \sim \frac{H}{\mu }$ is the scalar stiffness relating slip with shear stress change on a rectangular element^58^. The shear and normal stress changes at the secondary fault due to aseismic slip on the main fault are denoted as $\Delta \tau _{as}$ and $\Delta \sigma _{as}$, and are computed using discrete stiffness matrices relating slip and stress change on rectangular fault elements in elastic half space^58^. Δσ_S_ denotes normal stress change due to slip on the secondary fault.

In our simulation, the number of spring sliders used is smaller than the observed number of microseismic events by a factor of 15 ($1 \times 10^3$ spring sliders vs. $\sim 1.5 \times 10^{4}$ events by day 18), for efficient modeling. During the simulation, only $\sim 20 \%$ of spring sliders are triggered (see Workflow for spatial extent of seismicity). Therefore, this approach is likely more than 15 times more efficient than simulating all observed seismicity.

### Theoretical model for spatial extent of seismicity

Here we derive a theoretical model for the spatial extent of direct fault weakening induced seismicity. Assume each secondary fault initially has the same shear stress, $\tau _0$, and effective normal stress, $\sigma _0^{'}$, the strength drop, due to pore pressure change, $\Delta p$, required to generate seismicity can be approximated as:

10 $$\begin{aligned} \Delta \tau = f_0 \sigma _0^{'} - \tau _0 = f_0 \Delta p (r, t, dz) \end{aligned}$$

where $f_0$ is the initial frictional coefficient, and $\Delta p (r, t, dz)$ is the pressure perturbation at vertical distance *dz* away from the boundary of the fault zone. We utilize the following analytical solution for pore pressure change at secondary faults due to step function injection on the main fault:

11 $$\begin{aligned} \Delta p(r, t, dz) = - \frac{Q_0 \eta }{4 \pi k w} E_i \left( -\frac{1}{4} \frac{\phi \eta \beta r^2}{k t}\right) \exp \left( -\frac{dz^2}{2 \Sigma ^2}\right) \end{aligned}$$

where the expression in front of the exponential is the well-known Theis solution^49^ for injection into a 3D, infinite fault of a finite width. The exponential term corresponds to off-fault pressure diffusion as discussed in the main text. $\Sigma$ is the standard deviation for the decay of pore pressure perturbation as a function of vertical distance away from the fault boundaries (assuming Gaussian distribution). Equating Eqs. (10) and (11) (yielding a transcendental equation), we numerically solve for the maximum radial distance from the injection well, $R = f(t, dz)$, where seismicity is permissible.

## Data Availability

Code used for simulation is available online through Zenodo (https://doi.org/10.5281/zenodo.7261939). Injection volume rate history, wellhead pressure history, and seismic catalogue are available through EPISODES Platform (https://tcs.ah-epos.eu/#episode:COOPER_BASIN).
